# Staging System to Predict the Risk of Relapse in Multiple Myeloma Patients Undergoing Autologous Stem Cell Transplantation

**DOI:** 10.3389/fonc.2019.00633

**Published:** 2019-07-12

**Authors:** Chitrita Goswami, Sarita Poonia, Lalit Kumar, Debarka Sengupta

**Affiliations:** ^1^Department of Computer Science and Engineering, Indraprastha Institute of Information Technology, New Delhi, India; ^2^Department of Computational Biology, Indraprastha Institute of Information Technology, New Delhi, India; ^3^Department of Medical Oncology, All India Institute of Medical Sciences, New Delhi, India; ^4^Infosys Center for Artificial Intelligence, Indraprastha Institute of Information Technology, New Delhi, India

**Keywords:** multiple myeloma, autologous stem cell transplantation, risk of relapse, multivariate survival analysis, spectral clustering, fast and frugal tree

## Abstract

Over the last decade autologous stem cell transplantation (ASCT) has emerged as the standard of care in the management of Multiple Myeloma (MM). However, the cases of early relapse (within 36 months) after the stem cell rescue remains a significant challenge. For a lot of practical purposes, it is crucial to identify whether a patient undergoing ASCT falls into the high-risk group (likely to relapse within 36 months) or a low risk one. Our analysis showed that existing MM staging systems (International Staging System or ISS and Durie Salmon Staging or DSS) are not sufficient to discriminate between the risk groups significantly. To address this, we gathered a total of 39 clinical and laboratory parameters of 347 patients from the Department of Medical Oncology of All India Institute of Medical Sciences (AIIMS). We employed a stacked machine learning model consisting spectral clustering and Fast and Frugal Tree (FFT) technique to come up with a 3-factor multivariate 2-stage staging scheme, which turns out to be extremely decisive about the outcome of the stem cell rescue. Our model comes up with a three-factor (1. if patients has relapsed following remission, 2. response to induction, 3. pre-transplant Glomerular Filtration Rate or GFR) staging scheme. The resulting model stratifies patients into high-risk and low-risk groups with markedly distinct progression-free (median survival—24 months vs. 91 months) and overall survival (median survival—51 months vs. 135 months) patterns.

## 1. Introduction

Multiple Myeloma (MM) is a cancer of plasma cells. Clonal expansion of malignant plasma cells in bone marrow and the presence of monoclonal protein (M-protein) in blood and urine are the disease hallmarks (1, 2). MM is the second most common of all hematological cancer after non-Hodgkin lymphoma (3). It is responsible for 15–20% of the deaths attributable to the hematological malignancies and about two percent of all cancer-related deaths (4). Worldwide, MM affects 1–5 per 100 thousand individuals per year with a higher number of cases in western countries (5, 6). In a global, longitudinal study conducted concerning 32 cancer types, MM jumped from 23rd place in 2005 to 21st place in 2015 (5). In the United States, 1 in 132 is at risk of developing this disease in his lifetime. In 2018, estimated new cases of multiple myeloma will be 30,770, and an estimated 12,770 people will die of this disease (7). A study suggests that the incidence of multiple myeloma in black people is double as compared to white people (8).

The 5-year survival rate for people with multiple myeloma has steadily increased over the past two decades, During 1975–1977 the 5-year survival rate for MM was 25%. It became 47% during 2004–2010 (9). Currently, we are observing a 5-year survival rate of 50% (10). This increased survival rate is a result of advancement in the treatment of the disease (11, 12). Introduction of novel agents over alkylating agents in induction therapy and high-dose chemotherapy followed by autologous stem cell transplantation (ASCT) considerably improved the survival of multiple myeloma patients in the past several years (13–17). ASCT in the era of novel agents plays a crucial role in the management of younger MM patients. Patients receiving upfront ASCT have been found to have improved progression-free survival (PFS) and overall survival (OS) compared to patients receiving the conventional chemotherapy (CC) (18–20).

Presently, at the beginning of the treatment, all the patients are treated with induction therapy for 4–6 months using a combination of novel agents such as proteasome inhibitors (bortezomib), immune modulators (lenalidomide, thalidomide), and dexamethasone. After induction therapy, patients younger than 65 years of age (2) are advised to undergo further treatment of high dose melphalan, followed by ASCT. Further, maintenance therapy is administered to the patients for 1–2 years using lenalidomide/thalidomide, lenalidomide, or bortezomib (2, 14, 21). A number of randomized (22) and non-randomized trials, meta-analyses, and population-based studies have provided evidence in favor of the efficacy of this regime, measured in terms of high response rates, improved OS and PFS (17, 23). Despite the promise, some patients relapse within two years of the graft (24). It is therefore important to develop predictive models to identify patients who are at high risk of early relapse.

To address this issue, we analyzed clinical data of 253 multiple myeloma patients [(median age—52 years, 166 males, 87 females), (between August, 2005 to December, 2016)], who were treated at the Department of Medical Oncology of All India Institute of Medical Sciences (AIIMS). We used Fast and Frugal Tree (FFT) for constructing a tree-based model for stratifying patients into either a high-risk or a low-risk group. The tree-based model included factors concerning: 1. If the relapse occurs after remission, 2. response to induction therapy, and 3. (pre-transplant) Glomerular Filtration Rate (GFR), which are commonly available prior to the transplant. Our 2-stage staging scheme yielded significantly distinct survival pattern between the risk groups both for progression free and overall survival.

## 2. Materials and Methods

### 2.1. Patients

Between April 1990 and December 2016, 347 patients with MM underwent ASCT at the Department of Medical Oncology of All India Institute of Medical Sciences (AIIMS). Written consent was obtained from all patients for the study. The study has been approved by the Institute of Ethics Committee, All India Institute of Medical Sciences(AIIMS) with the approval number: IEC-523/05.10.2018.

### 2.2. Transplant Protocol

Initially, all patients were reviewed in the weekly Bone Marrow (BM) Transplant Clinic in which the associated risks and benefits of bone marrow transplantation were explained to the patients and their family members. Pre-transplant evaluation included a detailed history, physical examination, staging according to the Durie and Salmon (DSS) (25) and the International Staging System (ISS) (26). Details of previous treatment were recorded. The pre-transplant investigations included hemoglobin, total and differential count, renal and liver function tests, bone marrow examination, skeletal survey, and serum and urine electrophoresis, immune-fixation studies, serum β-2 microglobulin, and quantitative immunoglobulin levels. Written informed consent was obtained. Regimen-related toxicity was defined as per the Seattle criteria (27).

The source of stem cells in most patients was granulocyte colony-stimulating factor (G-CSF) mobilized peripheral blood stem cells. Cyclophosphamide mobilized peripheral blood stem cells were used for stem cell harvesting in <10 patients. Even fewer patients had their stem cells harvested from bone marrow. The trypan blue dye exclusion test determined the viability of cells (28).

Induction therapy goes on for 4–5 months and usually consists of 4–6 cycles. The patients are treated with a combination of novel agents, e.g., immune modulators (thalidomide, lenalidomide), proteasome inhibitors (bortezomib), and dexamethasone, following which patients are treated with high dose melphalan (29).

The myeloablative conditioning regimen consisted of melphalan dosage 150−225*mg*/*m*^2^ (218 patients, 86.2%) slow i.v. push on day 1 of transplantation followed by forced alkaline diuresis. Melphalan dosage of ≤ 150*mg*/*m*^2^ (35 patients, 13.8%) was given to patients with renal insufficiency [eGFR <40*ml*/*min*/1.73*m*^2^, according to MDRD formula (30)] at the time of transplantation. With the change in melphalan dosage, no significant difference in the outcome of PFS and OS was observed (Table S4; Figure S6). This is concurrent with previous literature (31).

Stem cells were transfused intravenously (i.v.) 24 h after conditioning patients with high-dose of melphalan. 5μ*g*/*kg* stem cells administered subcutaneously daily, including on day 0, 12 h after stem cell infusion and onwards until engraftment. Patients were treated in isolation rooms and reverse barrier nursing was practized.

### 2.3. Data Pre-processing

We used 39 variables (Table S1) from the clinical and laboratory data for the univariate analysis whereas 36 of them were used for the multivariate analysis. We ensured information related to these variables are typically available in the pre-transplant phase. Some of the variables had missing values (Figure S1), which were subjected to missing value imputation using an R package implementing MICE, a widely used algorithm for this purpose (32). Categorical variables were transformed into numerical ones with the use of one hot encoding. This is essential for the machine learning based algorithms to work.

### 2.4. Univariate Analysis

Associations of the individual factors w.r.t. OS and PFS were analyzed using the widely used Kaplan Meier's survival analysis technique (33). Categorical variables were grouped by categories, whereas the numerical variables (23 out of 39) were subjected to univariate K-Means (34) for exploring groups. For simplicity, the number of clusters was set 2 for each case. A cut-off value was generated based on the highest observed value of the cluster comprising the smaller values. If the highest observed value is *C*, the associated ranges are ≤ *C* and > *C*.

### 2.5. Multivariate Analysis

Typically, variables in combination hold promise for a more nuanced predictive model. Predictive modeling involves training of the model, followed by validation. When the sample size is small, taking out data-points for validation turns out to be detrimental as it weakens the model training. On the flip side, training a model on the entire data is usually suspect for model overfitting.

**Figure 1:**
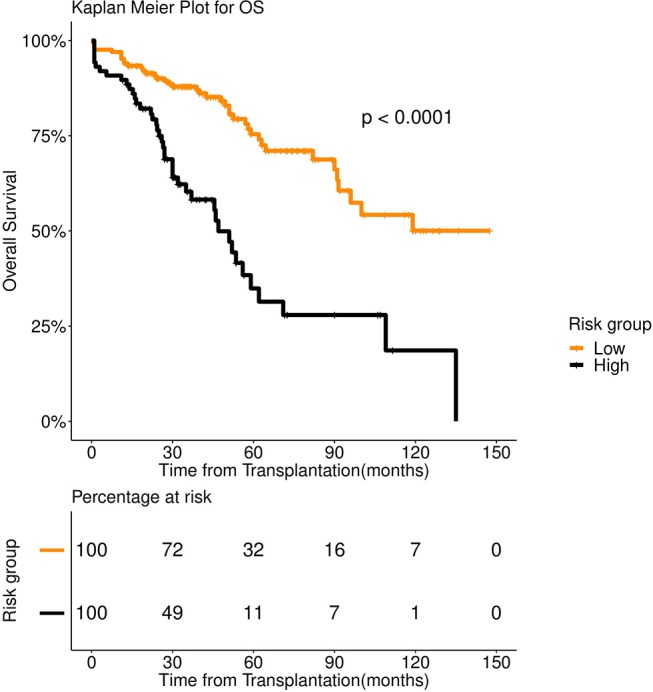
Overall survival (OS) in 253 patients with multiple myeloma stratified by spectral clustering. Median OS was more than 90 months for low risk group (number of patients = 166, events = 40) (orange color), whereas it was 47 months for high risk group (number of patients = 87, events = 42) (black color).

**Figure 2:**
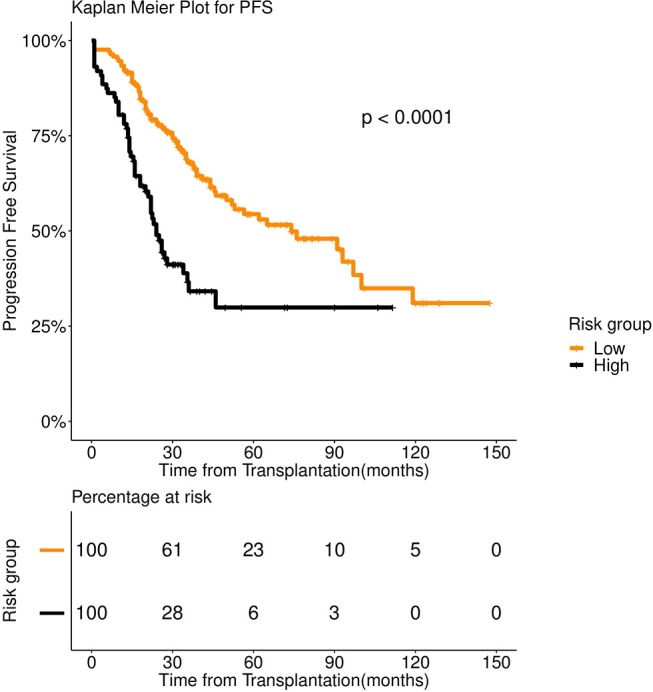
Progression Free survival (PFS) in 253 patients with multiple myeloma stratified by spectral clustering. Median PFS was 74 months for low risk group (number of patients = 166, events = 70) (orange color), whereas it was 24 months for high risk group (number of patients = 87, events = 50) (black color).

We bypassed this problem by developing a two-pronged learning approach. We first grouped the patients using spectral clustering (35). For this, we constructed an adjacency matrix spanning the data points (patients) by computing the Hamming distance of each point pair. Continuous variables were considered in their binary form for the distance calculation. Principal Component Analysis (PCA) was performed on the distance matrix. Principal Components entailing 95% of the Eigen energy were subjected to spectral clustering. Two clusters thus obtained showed distinct survival patterns both for OS and PFS (Figures 1, 2). We treated the clusters as high-risk and low-risk groups. To aid clinical decision making, we fitted a Fast and Frugal Tree (FFT) (36) for accurate prediction of risk groups. An FFT is a simpler version of a decision tree (37). The most striking feature of FFT is that unlike decision tree it is usually simple enough for a human mind to memorize. FFTs have been shown to perform competitively with random forest (37).

FFT, being conspicuously simple, does not warrant overfitting. Therefore, we refrained from independent validation of tree performance. On the first pass, we trained a 2-class FFT, while treating the cluster identities as the labels for the patients under study. We then subjected the samples to the trained FFT to re-calibrate the labels.

As evident from the accuracy on the training data (84.9%), the FFT managed to model the clusters. In fact, the slight modifications of the labels caused an increase in the median survival of the low-risk group in case of PFS (91 months instead of 74 months), while the median survival of the high-risk group remained unaltered (24 months).

## 3. Results

### 3.1. Patient Characteristics

Figure S2 depicts year wise distribution of patient frequencies. Patients were administered various drug combinations during induction therapy. The drug regimen for induction therapy has undergone significant changes since the 90's, usage of novel agents being the current trend. VAD regimen [Vincristine (V), Doxorubicin (A), Peroral Dexamethasone (D)] was administered to 72 patients (between April 1990 and March 2005). Alkylating agents were given to only 22 patients (between July 1997 and March 2014). This trend is illustrated in Figure S3. As expected, novel agents yielded improved survival as compared to VAD and Alkylating agents (Figure S4). Two hundred and fifty three patients were treated with novel agents from August 2005 to December 2016. For our study, we considered only the 253 patients who were treated with various novel agents (Table S2) during induction therapy, since that has been the most prevalent mode of treatment during the last decade. No significant difference was observed in the survival trends across the novel-agents (Figure S5). No patient was lost in follow up. Follow up was done till 30th November 2017 (date of censor). For patients treated with novel agents, 8 out of 253 had undergone dialysis. Post-transplant, only one of these 8 patients had undergone elective dialysis. The patient subsequently underwent renal transplant as well and continued to be disease-free for more than 2 years. Some important patient characteristics are shown in (Table 1).

**Table 1:** Patient characteristics.

**Characteristics**	**Number of patients out of 253**
**Median age**	52
**Gender**	166 males, 87 females
**Relapsed after remission, then transplant**	69
**Diabetic**	107
**Presence of extramedullary disease**	57
**Renal condition**	
Required dialysis	8
**ISS stage during diagnosis**	
Stage-I	67
Stage-II	94
Stage-III	92
**Line of induction therapy**	
One line	174
Two line	54
Three line	19
>Three line	6
**Immonoglobulin type**	
IgG-Kappa	101
IgG-Lambda	43
IgA-Kappa	26
IgA-Lambda	15
Kappa	45
Lambda	23

### 3.2. Factors Affecting Response to Transplant

We performed Kaplan Meier's survival analysis for the individual factors to determine the ones that have prognostic value. Out of a total of 39 factors (Table S1), 23 were numerical (pre-transplant M-protein level, pre-transplant GFR etc.). We grouped patients based on each numerical feature using univariate K-means (Methods). We tracked both overall and progression-free survival for each of the factors. Factors that displayed remarkable prognostic value for both OS and PFS were: 1. If patient relapsed following remission (*P* < 0.001 for both OS and PFS), 2. the number of regimens used pretransplant (*P* < 0.001 for both OS and PFS), 3. serum albumin level (*P* < 0.001 for both OS and PFS) and pre-transplant M-protein level (*P* = 0.0018 for PFS and *P* = 0.002 for OS). Interestingly, response to induction therapy (Table S5) (*P* = 0.0015) showed relatively greater prognostic merit for PFS as compared to OS (*P* = 0.012). Interesting ISS staging appeared minimally predictive for PFS. Important outcomes of the univariate analyses are captured in (Table 2). We found K-Means based grouping to be scientific and approximately aligned with previous findings. For instance, we obtained a cut-off of 3.5 *g*/*dL* for serum albumin level, which we cross-referenced with a previous study that linked serum albumin level ≤ 3.5 *g*/*dL* with higher mortality (38).

**Table 2:** Prognostic power of the individual factors.

**Factor**		***n***	**Progression free survival**					**Overall survival**				
			**Events**	**Median**	***P*****-Value**			**Events**	**Median**	***P*****-Value**		
Relapse status	Non relapsed, i.e., First line transplantation	184	74	76	<0.001			43	135	<0.001		
	Relapsed, then salvage re-induction therapy, followed by ASCT	69	46	22				39	37			
Induction line	1	174	69	76	<0.001			41	135	<0.001		
	>1	79	51	24				41	47			
Albumin	< =3.5	103	58	34	<0.001			45	56	<0.001		
	>3.5	150	62	76				37	119			
Response induction	CR + VGPR	142	57	91	0.0015			36	100	0.012		
	Others	111	63	35				46	63			
PretranplantM spike	< =0.25 g/dL	175	73	56.5	0.0018			43	>90	0.002		
	>0.25 g/dL	78	47	28.0				39	62			
ISS	ISS-I	67	29	76		ISS-I	ISS-II	16	130		ISS-I	ISS-II
	ISS-II	94	48	44	ISS-II	0.2	−	34	91.5	ISS-II	0.0556	−
	ISS-III	92	43	36	ISS-III	0.15	0.42	32	62	ISS-III	0.0042	0.1129
EMD	Present	57	32	35.5	0.042			25	57	0.014		
	Absent	196	88	53				57	100			
Hb (g/dl)	< =8.3	82	44	34	0.046			30	96	0.029		
	>8.3	171	76	62				52	100			
Serum M spike	< =1.91 g/dL	95	37	76	0.077			22	>100	0.071		
	>1.91 g/dL	158	83	39				60	90			
GFR (mL/min/1.73 m^2)	< =59.45	158	72	56.5	0.21			47	96	0.06		
	>59.45	95	48	44				35	64.5			
Beta2microglobulin (mcg/L)	< =4.2 mcg/mL	129	59	62	0.11			37	135	0.0095		
	>4.2 mcg/mL	124	61	44				45	64			
Immunoglobulin Type	Kappa	172	79	53	0.41			51	109	0.076		
	Lambda	81	41	46				31	63			

### 3.3. Multi-Factor Survival Modeling

We found multiple variables to have an independent association with survival. Moreover, single variable risk stratification is of limited use for its restrictive nature. For instance, it may so turn out that a fraction of first line patients relapse soon after the graft. If we create a rather simplistic single factor staging scheme just based on the relapse (after remission) status, it may under-predict for those at risk. For multi-factor modeling exercise, a major hindrance is small sample size. Commonly used methods such as survival tree (39) requires a large number of samples to produce a meaningful model. For instance, the International Staging System (ISS) was built on clinical and laboratory data of about 10,000 patients (26).

We first examined the heterogeneity in the patient population using spectral clustering (Methods). All 39 pre-transplant variables were used for this. We obtained two clusters that showed a stark difference in survival pattern both for PFS (*P* < 0.001) and OS (*P* < 0.001). See Figures 1, 2 for the associated Kaplan Meier analysis. We marked the patient-groups mirrored by the clusters as high-risk and low-risk depending on their survival trend. The high-risk group consisted of 34% of the patients with a median progression-free survival of 24 months. On the contrary, the low-risk group consisted of 66% of the patients with a median progression-free survival of 74 months (Figure 2). While clusters are useful to unravel patient heterogeneity, they don't augment clinical decision making. To this end, we used a novel iterative approach for constructing a Fast and Frugal Tree (FFT) that effectively models the clusters (Methods). The tree is meant for mapping any patient to one of the risk groups depending on his/her characteristics.

**Figure 3:**
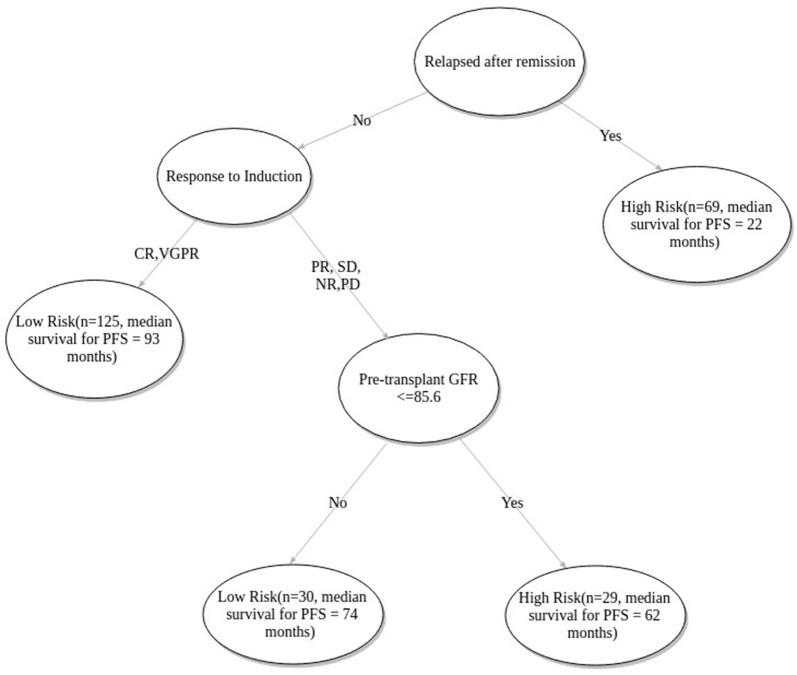
Fast-and-frugal tree based staging scheme for patients undergoing ASCT. CR, Complete Response; VGPR, Very Good Partial Response; PR, Partial Response; NR, No Response; SD, Stable Disease; PD, Progressive disease.

**Figure 4:**
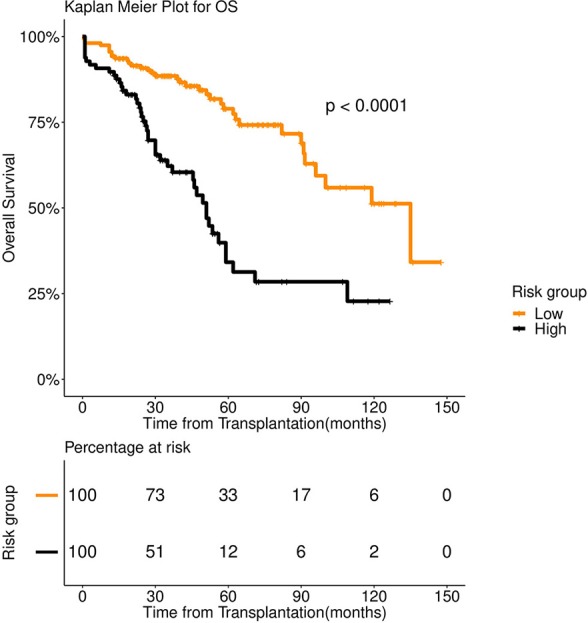
Overall survival (OS) in 253 patients with multiple myeloma stratified by FFT rules. Median OS was 135 months for low risk group (number of patients = 156, events = 36) (orange color), whereas it was 51 months for high risk group (number of patients = 97, events = 58) (black color).

**Figure 5:**
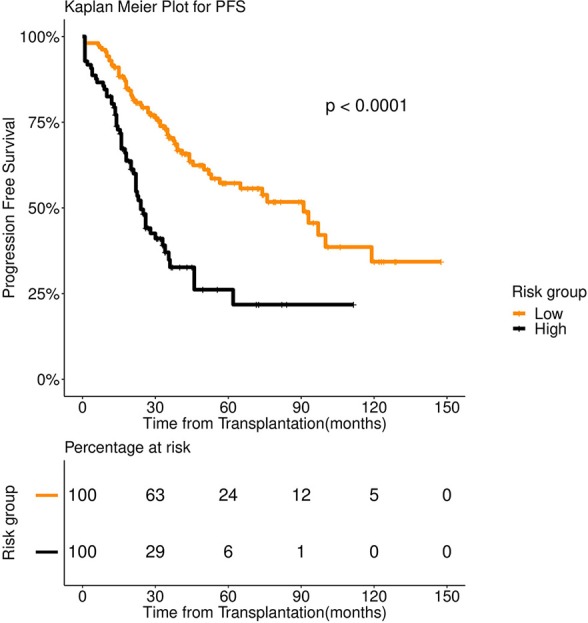
Progression Free survival (PFS) in 253 patients with multiple myeloma stratified by FFT rules. Median PFS was 91 months for low risk group (number of patients = 156, events = 62) (orange color), whereas it was 24 months for high risk group (number of patients = 97, events = 58) (black color).

FFT based modeling offered a simple, 3-factor decision tree that predicts the risk category of a patient. It's similar to a staging scheme. Variables elected by the final FFT included: 1. If patient relapsed following remission, 2. response to induction, and 3. pre-transplant GFR (Figure 3). Subjecting patients to the FFT showed better discrimination in survival patterns across the re-calibrated high-risk and low-risk groups (Methods). While the median progression-free survival of the high-risk group remained unchanged (24 months), for the low-risk group, we obtained a median survival of 91 months (see Figures 4, 5) for the KM analyses for OS and PFS). Notably, we found the risk-groups to have partial concordance with the variables having independent prognostic value (Table S3).

We excluded ISS and DSS from the scope of the multi-variate modeling since these are dependent on variables which already exist in our data. We excluded the variable depicting the number of induction line since it's highly correlated with disease relapse (after remission) status. Its inclusion gives rise model overfitting.

### 3.4. Prognostic Value of Alternative Staging Systems

We evaluated the prognostic value of the existing, widely practiced staging systems—Durie Salmon Staging (DSS) (25) and International Staging Systems (ISS) (26). DSS relies on hemoglobin concentration, level of blood calcium, the presence of bone lesions, M protein level in urine and blood and kidney function level to predict the extent of the disease. ISS, on the other hand, uses albumin and Beta-2-microglobulin levels for staging patients with MM. One must note that DSS and ISS are not meant for predicting the outcome of stem cell rescue. We found DSS to be an extremely weak predictor of the ASCT outcome. We observed some association across ISS-I—ISS-II(*P* = 0.0556), and ISS-I—ISS-III (*P* = 0.0042) in case of OS. For PFS, ISS staging turned out to be a weak predictor [ISS-I—ISS-II (*P* = 0.2), and ISS-I—ISS-III (*P* = 0.15)].

## 4. Discussion and Conclusion

Treatment of MM has improved markedly in the past two decades. A lot of this success is attributable to Autologous Stem Cell Transplantation (ASCT), which, over the past decade, has emerged as the standard of care for patients aged below 65 years (14, 17). In a retrospective analysis, we noted that the existing staging schemes [ISS (26) and DSS (25)] are of limited use due to their poor correlation with the graft outcome. This inspired us to explore the potential of multivariate modeling of the outcome of stem cell rescue in MM.

We used clinical and lab data of 253 patients who have been treated with novel agents and undergone ASCT at AIIMS between 2005 and 2016. Due to the small sample size, we developed a new machine learning approach that's minimally susceptible to the problem of model overfitting. We showed that a simple, 3-variable [If relapse occurs after remission, response to induction and (pre transplant) GFR] decision tree can serve as a staging scheme that maps each patient to one of the two (high and low) risk groups, with markedly distinct survival patterns for both overall and progression-free survival.

As per the proposed tree based model patients with relapsed disease (following remission) were predicted under the high-risk category. Median PFS of relapsed patients is 22 months compared to that of first-line patients which is 76 months (Table 2). Patients with relapsed MM do better with ASCT but relative to other patients (non-relapse) their survival is poor (40, 41). The model correctly identifies the state of relapse as the key factor for risk prediction. As previous literature suggests, 90% of the patients exhibiting complete response to induction therapy, also exhibit complete response to ASCT. In case of very good partial response during induction, the corresponding complete response is 72% (29). The patients who do not show complete response and very good partial response are likely to relapse quicker (42). The model correctly identifies this variable as the next important factor for relapse prediction. Renal functioning is an important factor for multiple myeloma patients since renal insufficiency is positively correlated with increased mortality (43). GFR grade is defined as ≥90, ≥60−89, ≥30−59, 15−29, ≤ 15*ml*/*min*/1.73*m*^2^ as per the standard criteria (44). The model predicted cutoff of 85.6 closely approximated the recommended cutoff for stage 1 i.e., GFR ≥90*ml*/*min*/1.73*m*^2^.

Many studies suggest age an important predictive factor for ASCT outcome (29, 45). We observed that patients regardless of age, appear to benefit from ASCT. This has been seconded by previous studies (46), as well as in our data. The median age of patients treated with novel agents is 52 years. No significant differences (*P* = 0.31 for PFS and 0.36 for OS) were observed between the two groups (<=52 and >52 yrs). As a result, age has not been picked up by the decision tree among the top influencers.

The model we obtained also highlights the importance of multivariate analysis. Pre-transplant GFR, independently, did not emerge as an important prognostic factor (Table 2). However, when combined with the relapse (following remission) status and response to induction therapy it led to a more nuanced stratification of the patients into the risk categories. Despite no stark difference in the median PFS (high-Risk—62 months; low-risk—74 months), patients subjected to the GFR mediated bifurcation showed a significant difference in the rates of 5-year survival rate (37 vs. 14%) (Figure S7).

Due to data paucity, we did not apply excessive inclusion/exclusion criteria besides considering only those patients who were treated with novel agents. Apparently, our multivariate model discerned the variable outcomes between newly diagnosed and relapsed (after remission) patient groups using a limited number of pre-transplant clinical variables. This also serves as a testimony for the model's inherent ability to accurately predict graft outcome for diverse patient strata. The proposed tree based model labels relapse after remission cases as “High-risk” (Figure 3).

To test the ubiquity of the newly proposed staging scheme we employed additional data of patients, treated with VAD and alkylating agents. Application of our 3-factor rule sets stratified the mixed pool of patients into the high and low-risk categories. Kaplan Meier survival analysis yielded distinct (overall and progression-free) survival patterns (*P* < 0.0001 in both cases), following the trend observed on the patients treated with novel agents (Figures S8, S9).

A limitation of the study is the unavailability of cytogenetic/FISH data which is incorporated in the revised ISS system (47). The patient data is collected over a long period of time (2005–2017) and we did not have cytogenetic/FISH data for the initial period (till 2011). Another shortcoming of the current study is sample paucity. We plan to perform a multi-center follow-up study to ascertain the integrity of our staging scheme.

## Data Availability

The datasets generated for this study are available on request to the corresponding author.

## Ethics Statement

This study was carried out in accordance with the recommendations of Institute of Ethics Committee, All India Institute of Medical Sciences, with written informed consent from all subjects. All subjects gave written informed consent in accordance with the Declaration of Helsinki. The protocol was approved by the Institute of Ethics Committee, All India Institute of Medical Sciences.

## Author Contributions

DS and LK conceived the study. CG and SP developed the computational methods and conducted the various analyses under the supervision of DS. LK managed the patient data curation. All the authors discussed the results, co-wrote, and reviewed the manuscript.

### Conflict of Interest Statement

The authors declare that the research was conducted in the absence of any commercial or financial relationships that could be construed as a potential conflict of interest.

## References

[1] Kyle RA Rajkumar SV. Multiple myeloma. N Engl J Med. (2004) 351:1860–73. 10.1056/NEJMra041875 15509819

[2] Palumbo A Anderson K. Multiple myeloma. N Engl J Med. (2011) 364:1046–60. 10.1056/NEJMra1011442 21410373

[3] Kazandjian D. Multiple myeloma epidemiology and survival: a unique malignancy. In: Ahn IE Mailankody S, editors. Seminars in Oncology, Vol. 43. Elsevier (2016). p. 676–81. 28061985 10.1053/j.seminoncol.2016.11.004 PMC5283695

[4] Smith D Yong K Multiple myeloma. BMJ (2013) 346. 10.1136/bmj.f3863 23803862

[5] Fitzmaurice C Allen C Barber RM Barregard L Bhutta ZA Brenner H. Global, regional, and national cancer incidence, mortality, years of life lost, years lived with disability, and disability-adjusted life-years for 32 cancer groups, 1990 to 2015: a systematic analysis for the global burden of disease study. JAMA Oncol. (2017) 3:524–48. 10.1001/jamaoncol.2016.5688 27918777 PMC6103527

[6] Parkin DM Bray F Ferlay J Pisani P. Global cancer statistics, 2002. Cancer J Clin. (2005) 55:74–108. 10.3322/canjclin.55.2.74 15761078

[7] Siegel RL Miller KD Jemal A Cancer statistics, 2018. Cancer J Clin. 68:7–30. 10.3322/caac.21442 29313949

[8] Waxman AJ Mink PJ Devesa SS Anderson WF Weiss BM Kristinsson SY. Racial disparities in incidence and outcome in multiple myeloma: a population-based study. Blood. (2010) 116:5501–6. 10.1182/blood-2010-07-298760 20823456 PMC3031400

[9] Siegel RL Miller KD Jemal A Cancer statistics, 2015. Cancer J Clin. (2015) 65:5–29. 10.3322/caac.21254 25559415

[10] Wong R Tay J Economics of multiple myeloma. Blood. (2018) 132:4773 10.1182/blood-2018-99-110140

[11] MultipleMyelomaStatistics C Multiple Myeloma - Statistics. Cancer.Net (2018).

[12] Cancer.org Cancer Facts and Figures 2018. American Cancer Society (2018).

[13] Turesson I Velez R Kristinsson SY Landgren O. Patterns of improved survival in patients with multiple myeloma in the twenty-first century: a population-based study. J Clin Oncol. (2010) 28:830. 10.1200/JCO.2009.25.4177 20038719 PMC2834396

[14] Kumar SK Rajkumar SV Dispenzieri A Lacy MQ Hayman SR Buadi FK. Improved survival in multiple myeloma and the impact of novel therapies. Blood. (2008) 111:2516–20. 10.1182/blood-2007-10-116129 17975015 PMC2254544

[15] Gay F Oliva S Petrucci MT Conticello C Catalano L Corradini P. Chemotherapy plus lenalidomide versus autologous transplantation, followed by lenalidomide plus prednisone versus lenalidomide maintenance, in patients with multiple myeloma: a randomised, multicentre, phase 3 trial. Lancet Oncol. (2015) 16:1617–29. 10.1016/S1470-2045(15)00389-7 26596670

[16] Lehners N Becker N Benner A Pritsch M Löpprich M Mai EK. Analysis of long-term survival in multiple myeloma after first-line autologous stem cell transplantation: impact of clinical risk factors and sustained response. Cancer Med. (2018) 7:307–16. 10.1002/cam4.1283 29282899 PMC5806105

[17] Child JA Morgan GJ Davies FE Owen RG Bell SE Hawkins K. High-dose chemotherapy with hematopoietic stem-cell rescue for multiple myeloma. N Engl J Med. (2003) 348:1875–83. 10.1056/NEJMoa022340 12736280

[18] Mina R Larocca A Offidani M Bringhen S Caravita T Magarotto V Impact of complete response on survival with either autologous stem cell transplantation or conventional chemotherapy: results of a pooled analysis of 5 phase iii trials in newly diagnosed multiple myeloma patients. Blood. (2015) 126:927 Available online at: http://www.bloodjournal.org/content/126/23/927 (accessed July 02, 2019).26294714

[19] Gupta A Kumar L Dabkara D Gupta D Sharma O Sreeniwas V Multiple myeloma: autologous stem cell transplantation versus conventional chemotherapy–a retrospective age and stage matched analysis. J Clin Oncol. (2009) 27:7041 10.1200/jco.2009.27.15_suppl.7041

[20] Gay F Oliva S Petrucci MT Montefusco V Conticello C Musto P. Autologous transplant vs. oral chemotherapy and lenalidomide in newly diagnosed young myeloma patients: a pooled analysis. Leukemia (2017) 31:1727. 10.1038/leu.2016.381 28008174

[21] Röllig C. Knop S. Bornhäuser M. Multiple myeloma. Lancet. (2015) 385:2197–8. 10.1016/S0140-6736(14)60493-1 25540889

[22] Attal M Harousseau JL Stoppa AM Sotto JJ Fuzibet JG Rossi JF. A prospective, randomized trial of autologous bone marrow transplantation and chemotherapy in multiple myeloma. N Engl J Med. (1996) 335:91–7. 10.1056/NEJM199607113350204 8649495

[23] Fermand JP Katsahian S Divine M Leblond V Dreyfus F Macro M. High-dose therapy and autologous blood stem-cell transplantation compared with conventional treatment in myeloma patients aged 55 to 65 years: long-term results of a randomized control trial from the group myelome-autogreffe. J Clin Oncol. (2005) 23:9227–33. 10.1200/JCO.2005.03.0551 16275936

[24] Huang B Li J Lu J Xiao Y Zhao Y Huang H Scoring system for predicting risk of relapse in patients with multiple myeloma within two years after stem cell transplantation. Blood. (2017) 130(Suppl. 1):4531. Available online at: http://www.bloodjournal.org/content/130/Suppl_1/4531

[25] Durie BG Salmon SE. A clinical staging system for multiple myeloma correlation of measured myeloma cell mass with presenting clinical features, response to treatment, and survival. Cancer. (1975) 36:842–54. 10.1002/1097-0142(197509)36:3<842::AID-CNCR2820360303>3.0.CO;2-U 1182674

[26] Greipp PR Miguel JS Durie BG Crowley JJ Barlogie B Bladé J. International staging system for multiple myeloma. J Clin Oncol. (2005) 23:3412–20. 10.1200/JCO.2005.04.242 15809451

[27] Bearman SI Appelbaum F Buckner C Petersen F Fisher L Clift R. Regimen-related toxicity in patients undergoing bone marrow transplantation. J Clin Oncol. (1988) 6:1562–8. 10.1200/JCO.1988.6.10.1562 3049951

[28] Kumar L Ghosh J Ganessan P Gupta A Hariprasad R Kochupillai V. High-dose chemotherapy with autologous stem cell transplantation for multiple myeloma: what predicts the outcome? experience from a developing country. Bone Marrow Transplant. (2009) 43:81. 10.1038/bmt.2008.343 18978818

[29] Kumar L Boya RR Pai R Harish P Mookerjee A Sainath B. Autologous stem cell transplantation for multiple myeloma: long-term results. Natl Med J India (2016) 29:192. 28050994

[30] Levey AS Bosch JP Lewis JB Greene T Rogers N Roth D. A more accurate method to estimate glomerular filtration rate from serum creatinine: a new prediction equation. Ann Intern Med. (1999) 130:461–70. 10.7326/0003-4819-130-6-199903160-00002 10075613

[31] Ghilardi G Pabst T Jeker B Müller R Cairoli A Müller AM Melphalan dose in myeloma patients ≥65 years of age undergoing high-dose therapy and autologous stem cell transplantation: a multicentric observational registry study. Bone Marrow Transplant. (2019) 54:1029–37. 10.1038/s41409-018-0379-y 30390061

[32] Buuren SV Groothuis-Oudshoorn K mice: Multivariate imputation by chained equations in R. J Stat Softw. (2010) 45:1–68. 10.18637/jss.v045.i03

[33] Goel MK Khanna P Kishore J. Understanding survival analysis: Kaplan-meier estimate. Int J Ayurv Res. (2010) 1:274–8. 10.4103/0974-7788.76794 21455458 PMC3059453

[34] Song J Wang H Song MJ Package ‘ckmeans. 1d. dp': Optimal and Fast Univariate Clustering (2018).

[35] Ng AY Jordan MI Weiss Y On spectral clustering: analysis and an algorithm. In: Advances in Neural Information Processing Systems. Vancouver, BC (2002). p. 849–56.

[36] Todd PM Gigerenzer G. Précis of simple heuristics that make us smart. Behav Brain Sci. (2000) 23:727–41. 10.1017/S0140525X00003447 11301545

[37] Phillips ND Neth H Woike JK Gaissmaier W Fftrees: a toolbox to create, visualize, and evaluate fast-and-frugal decision trees. Judg Decis Making (2017) 12:344–68.

[38] Kim JE Yoo C Lee DH Kim SW Lee JS Suh C. Serum albumin level is a significant prognostic factor reflecting disease severity in symptomatic multiple myeloma. Ann Hematol. (2010) 89:391–7. 10.1007/s00277-009-0841-4 19844712

[39] Bou-Hamad I Larocque D Ben-Ameur H A review of survival trees. Stat Surveys (2011) 5:44–71. 10.1214/09-SS047

[40] Cornell R Kassim A. Evolving paradigms in the treatment of relapsed/refractory multiple myeloma: increased options and increased complexity. Bone Marrow Transplant. (2016) 51:479. 10.1038/bmt.2015.307 26726946 PMC4827007

[41] Zarabi SF Masih-Khan E Chen C Kukreti V Prica A Tiedemann R Results of salvage autologous stem cell transplantation (asct) for relapsed multiple myeloma (mm) in the era of novel agents: outcome of patients (pts) receiving prior bortezomib (btz)-based therapy. Blood. (2016) 128:5821.

[42] Harousseau JL Avet-Loiseau H Attal M Charbonnel C Garban F Hulin C. Achievement of at least very good partial response is a simple and robust prognostic factor in patients with multiple myeloma treated with high-dose therapy: long-term analysis of the ifm 99-02 and 99-04 trials. J Clin Oncol. (2009) 27:5720–6. 10.1200/JCO.2008.21.1060 19826130

[43] Heher EC Rennke HG Laubach JP Richardson PG. Kidney disease and multiple myeloma. Clin J Am Soc Nephrol. (2013) 8:2007–17. 10.2215/CJN.12231212 23868898 PMC3817918

[44] Levey AS Coresh J Balk E Kausz AT Levin A Steffes MW. National kidney foundation practice guidelines for chronic kidney disease: evaluation, classification, and stratification. Ann Intern Med. (2003) 139:137–47. 10.7326/0003-4819-139-2-200307150-00013 12859163

[45] Al Hamed R Bazarbachi AH Malard F Harousseau JL Mohty M. Current status of autologous stem cell transplantation for multiple myeloma. Blood Cancer J. (2019) 9:44. 10.1038/s41408-019-0205-9 30962422 PMC6453900

[46] LaPorte J Brown S Zhang X Bashey A Morris LE Holland HK Age related outcomes for multiple myeloma patients following autologous transplantation. Blood. (2014) 124:3968.

[47] Palumbo A Avet-Loiseau H Oliva S Lokhorst HM Goldschmidt H Rosinol L. Revised international staging system for multiple myeloma: a report from international myeloma working group. J Clin Oncol. (2015) 33:2863. 10.1200/JCO.2015.61.2267 26240224 PMC4846284

