# Supplementary material for: Staging System to Predict the Risk of Relapse in Multiple Myeloma Patients Undergoing Autologous Stem Cell Transplantation
Source: Front Oncol. 2019 Jul 12;9:633. doi: 10.3389/fonc.2019.00633 (PMC6640159; doi:10.3389/fonc.2019.00633)
Supplement: Supplementary file 1 [file Data_Sheet_1.PDF]

Supplementary Material

1 SUPPLEMENTARY TABLES AND FIGURES

1.1 Tables

Table S1. Variabe Information

Feature	Description
**Age**	Age of patient.
**Gender**	Gender of patient.
**Height**	Height of patient.
**Weight**	Weight of patient.
**Body surface area (BSA)**	Body surface area of patient.
**Body Mass Index (BMI)**	Body Mass Index of patient.
**Hb**	Hemoglobin level (g/dl) in blood when patient comes for first time.
**Creatinine**	Creatinine level in blood when patient comes for the first time.<br>High level creatinine indicates malfunctioning of kidney.
**Albumin**	Albumin level in blood when patient comes for the first time.<br>Lower serum albumin levels in multiple myeloma patients are<br>associated with clinical factors reflecting disease severity.
**Immunoglobulin type**	Immunoglobulin isotype in blood whether IgG-Kappa, IgG-Lambda, IgA-Kappa,<br>IgA-Lambda, Kappa, Lambda, IgMK, nonsecretory, IgD, IgM.
**Beta-2-microglobulin (B2M)**	The beta-2-microglobulin level in blood when the patient comes for the first time.<br>B2M is the most powerful prognostic predictor of multiple myeloma.<br>Lower serum albumin levels in multiple myeloma patients are associated with clinical factors<br>reflecting disease severity.(Greipp et al., 2005)
**Serum Calcium**	Serum calcium level in blood when the patient comes for the first time.<br>High calcium level in serum is related to advance multiple myeloma. It shows osteoclast.
**Extramedullary disease (EMD)**	Indicates whether patient has Extramedullary disease or not.<br>EMD is having myeloma tumors outside the bone marrow in soft tissues of body.
**Response to induction therapy**	Response to ASCT was assessed 6 weeks after transplant using the European Group for Blood and Bone Marrow<br>Transplantation (EBMT) criteria (BladÉ et al., 1998).<br>complete response, very good partial response,partial response, Stable disease, No respose, Progressive disease.
**Absolute Neutrophil Count (ANC)**	Neutrophil count when patient comes for the first time.
**Absolute lymphocyte count (ALC)**	Lymphocyte count when patient comes for the first time.
**Glomerular filtration Rate (GFR)**	Glomerular Filtration Rate when the patient comes for the first time.<br>It represents renal condition. Lower GFR is associated with clinical factors<br>reflecting disease severity.
**GFR Score**	GFR score is the categorical variable which shows how GFR changed over the induction therapy.
**GFR Grade**	Categorised GFR level: "1=>=90ml, 2= >=60 to 89, 3=>=30 to 59, 4=15-29, 5-<15ml".
**Primary (If relapse after remission)**	Patients are categorized into two categories.<br>1= patients who received treatment then went on to receive stem cell transplant.<br>2= patients relapse after remission,<br>then received salvage therapy followed by stem cell transplant.<br>Thus, *primary* indicates whether patient relapse after remission.
**Stem cell Harvest site**	Indicates whether stem cell harvest site is mobilized peripheral blood stem cells or bone marrow.
**BMPC**	Count of Bone Marrow Plasma cell while harvesting.
**Hypertension**	Indicates whether patient has hypertension or not.
**Diabetic**	Indicates whether patient has diabetes or not.
**Line of induction therapy**	Indicates the number of different types of regimens given in induction therapy.<br>One line consist 3 to 6 cycles (typically 4). One line means only one type of regimen<br>is given in all cycles. If a new combination of drugs is given than previous, then it will<br>consider in second line and so on.
**Number of cycles in 1st line treatment**	Regimens given in one cycle is combination of different drugs.<br>This variable tells us about number of cycles in one line of induction therapy.
**Radiation Therapy**	Tells us if patient received radiation therapy or not.<br>Radiation therapy is given after the preparation of stem cells to kill myeloma cells.
**Erythropoietin Treatment (Epo)**	Indicates whether patient received Erythropoietin treatment.<br>Epo treatment is associated with improved immunological functions.
**Dialysis**	Indicates whether receive dialysis.<br>Renal failure is the main reason of relapse in multiple myeloma patients<br>(Dimopoulos et al., 2010; Podymow et al., 2010).
**ISS**	International staging System.<br>It is used to calculate the three stages of multiple-myeloma by measuring serum albumin and beta-2-<br>microglobulin level . Stage I associated with less severity and Stage III associated with highest severity<br>(Greipp et al., 2005).
**DSS**	Durie-Salmon Staging System calculate staging of multiple myeloma patients by measuring blood calcium level,<br>hemoglobin level, M protein level and kidney function.<br>Higher the DSS stage associated with higher severity(Durie and Salmon, 1975).
**Platelet count**	Number of platelets when patient comes for the first time.
**Monoclonal protein level**	Monoclonal protein level in the blood when patient comes for the first time.<br>Higher level of monoclonal protein is directly associated with severity of disease.
**Symptom duration**	Duration of symptoms in months.
**Pre-transplant creatinine level**	Creatine level after induction therapy and before autologous stem cell transplantation.
**Pre-transplant GFR**	Glomerular filtration rate after induction therapy and before autologous stem cell transplantation.
**Pre-transplant M-protein level**	Serum M protein level after induction therapy and before autologous stem cell transplantation.
**Melphalan dose**	Dose of melphalan given as the conditioning regimen after collecting stem cells.
**CD34 cells number**	Number of CD34 cells in stem cell harvesting. It indicates the purity of stem cells in the sample.

Table S2. Table showing frequency of novel agents

**Novel Agent type**	**Number of patients**
**Two drug combination**	**180 (71.1%)**
Thalidomide/Dexamethasone	94
Lenalidomide/Dexamethasone	55
Bort/Dexa	31
**Three drug combination**	**71 (28.1%)**
Bortezomib/Thalidomide/Dexamethasone (VTD)	23
Bortezomib/Lenalidomide/Dexamethasone (VRD)	23
Bortezomib/Cyclophosphamide/Dexamethasone (VCD)	20
Cyclophosphamide/Thalidomide/Dexamethasone (CTD)	2
Bortezomib/Low-dose dexamethasone/Pegylated liposomal doxorubicin (PAD)	1
Melphalan/Prednisone/Thalidomide (MPT)	1
Bendamustine/Bortezomib/Dexamethasone (BVD)	1
**Four drug combination**	**2 (0.8%)**
Bortezomib/Thalidomide/Cyclophosphamide/Dexamethasone (VTCD)	2

Table S3. Correspondence of the risk groups with the variables that have independent prognostic value (see Table 1)

Factor	Low Risk (n=156)		High Risk (n=97)		P Value (PFS)
	No. of patients/Total no	%	No. of patients/Total no	%	
If relapse after remission (yes)	0/156	0	69/97	71	<0.001
Number of induction line used pre-transplant (one time)	143/156	91.6	0/97	0	<0.001
Serum albumin level (>= 3.5 g/dL)	97/156	62.1	53/97	54.6	<0.001
Response to induction therapy (CR, VGPR)	125/156	80.1	0/97	0	0.0015
Pre-transplant M-spike (>=2.5 g/dL)	28/156	17.9	50/97	51.5	0.0018

Table S4. Distribution of Melphalan dosage

Melphalan dose	No. of patients (%)
**150** mg/m^2	35 (13.8%)
**151-180** mg/m^2	130 (51.4%)
**181 - 200** mg/m^2	85 (33.6%)
**200 - 225** mg/m^2	3 (1.2%)

Table S5. Responses to induction therapy

Response to Induction	Description
**Complete Response (CR)**	Absence of M protein in serum and urine by immunofixation with 5% or fewer plasma cells on bone marrow aspiration maintained for at least six weeks, with no increase of lytic bone lesions and, the disappearance of soft tissue plasmacytomas (Durie et al. (2006)).
**Very Good Partial Response (VGPR)**	Serum and urine M (monoclonal) component are detectable by immunofixation but not on electrophoresis. OR, There is 90% reduction in serum M protein and urine M protein $<$100 mg per 24 hours (Durie et al. (2006)).
**Partial Response (PR)**	Serum M-protein reduces by $\geq$50% and, 24-h urinary M-protein reduces by $\geq$90% or to $<$ 200mg per 24h. When serum and urine M-protein cannot be measured, $\geq$50% reduction in the difference between involved and uninvolved FLC levels is needed along with serum and urine M-protein, if serum free light assay is also unmeasurable, $\geq$50% decrease in plasma cells is requisite, provided baseline bone marrow plasma cell percentage was $\geq$30%. If bone marrow and plasma cell is present at baseline, another requisite is $\geq$50% decrease in the size of soft tissue plasmacytomas. (Durie et al. (2006)).
**Progressive Disease (PD)**	Progressive disease (PD) was defined as any or more of the following:<br>**(i)** Increase of $\geq$25% from baseline Serum M-component and/or the absolute increase must be $\geq$ 0.5 g/dl.<br>**(ii)** An absolute increase of $>$200 mg of urinary M protein in 24 hours.<br>**(iii)** A new bone lesion or plasmacytoma.<br>**(iv)** an increase in the size of such lesions.<br>**(v)** The development of hypercalcaemia (serum calcium level $>$11.5 mg/dl).<br>**(vi)** Absolute Bone marrow plasma cell percentage should be $\geq$10%.<br>**(vii)** For patients without measurable serum and urine M-protein levels, the absolute increase in the difference between involved and uninvolved FLC levels should be $>$10mg/dl. (Durie et al. (2006)).
**Stable Disease (SD)**	When patient can't be categorized into CR,VGPR,PR or progressive disease. (Durie et al. (2006)).

1.2 Figures

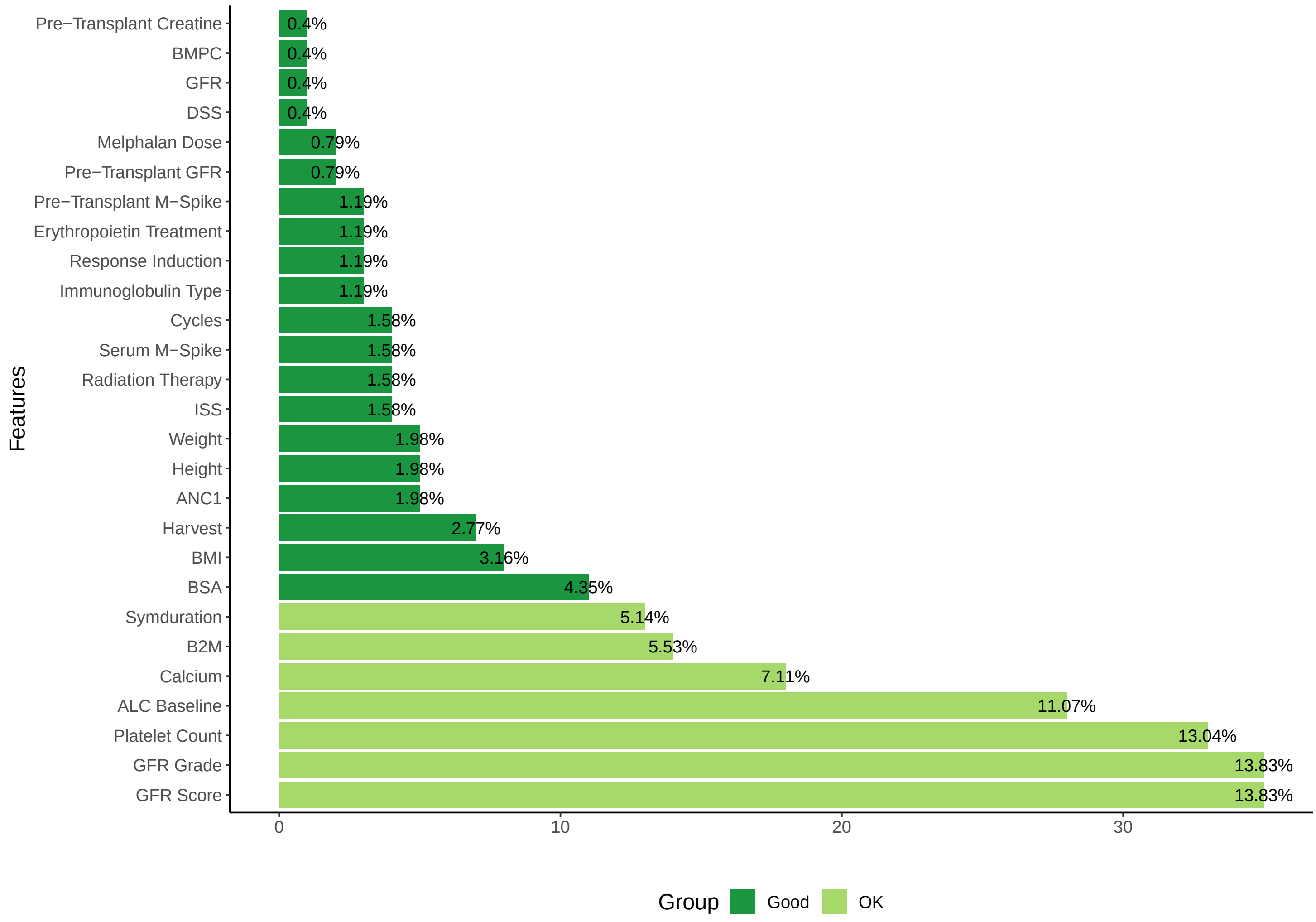

Figure S1: Percentage of missing values

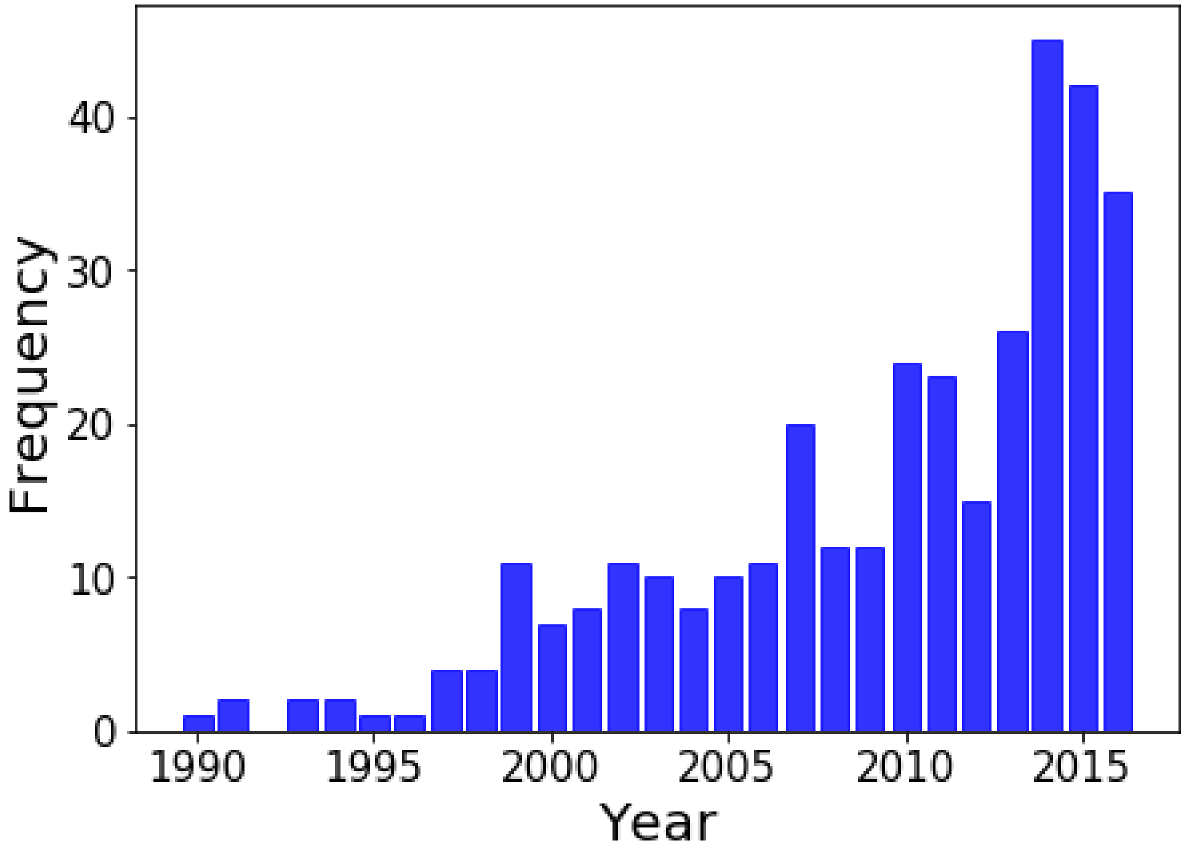

Figure S2: Patient distribution over the years

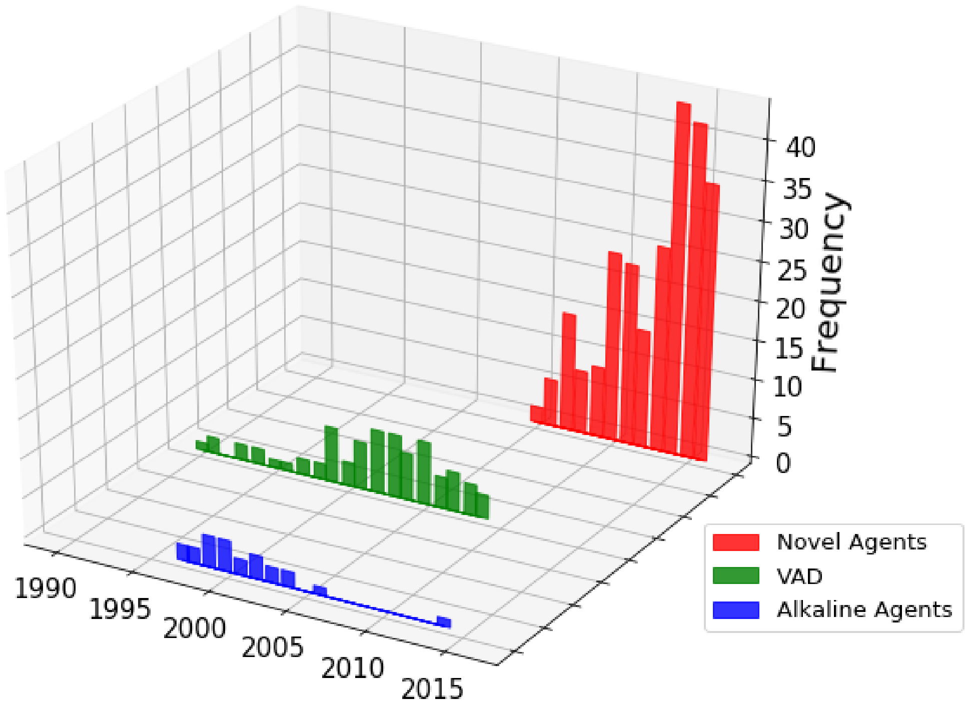

Figure S3: Changing trend of drugs used during induction regimen. Novel Agents - 253 (72.91%), VAD - 73 (21.04%), Alkylating agents - 21 (6.05%)

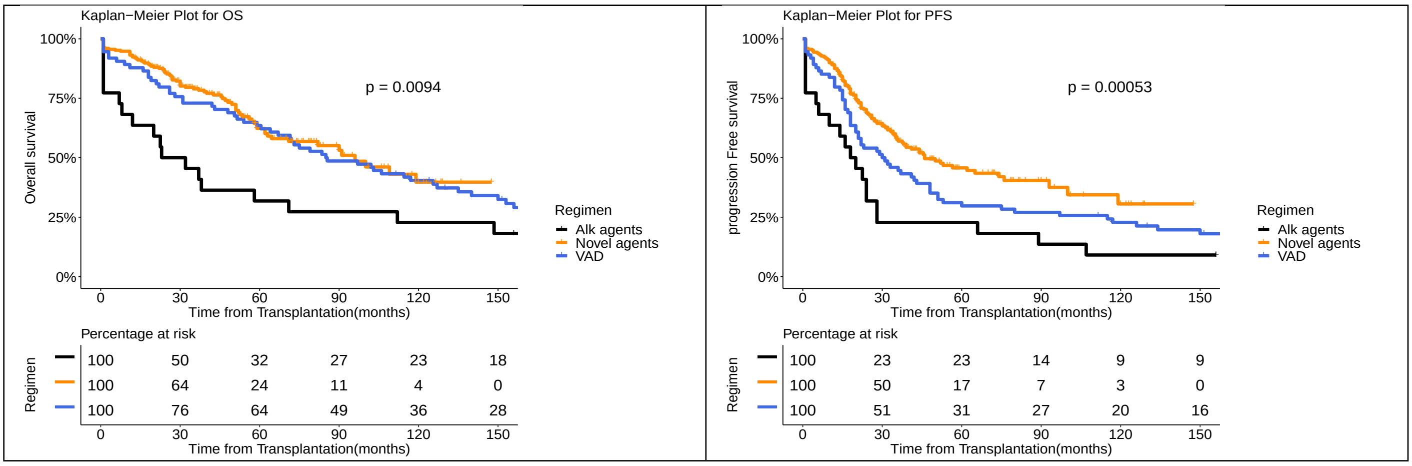

Figure S4: OS and PFS comparison of Alakine, VAD, Novel Agents

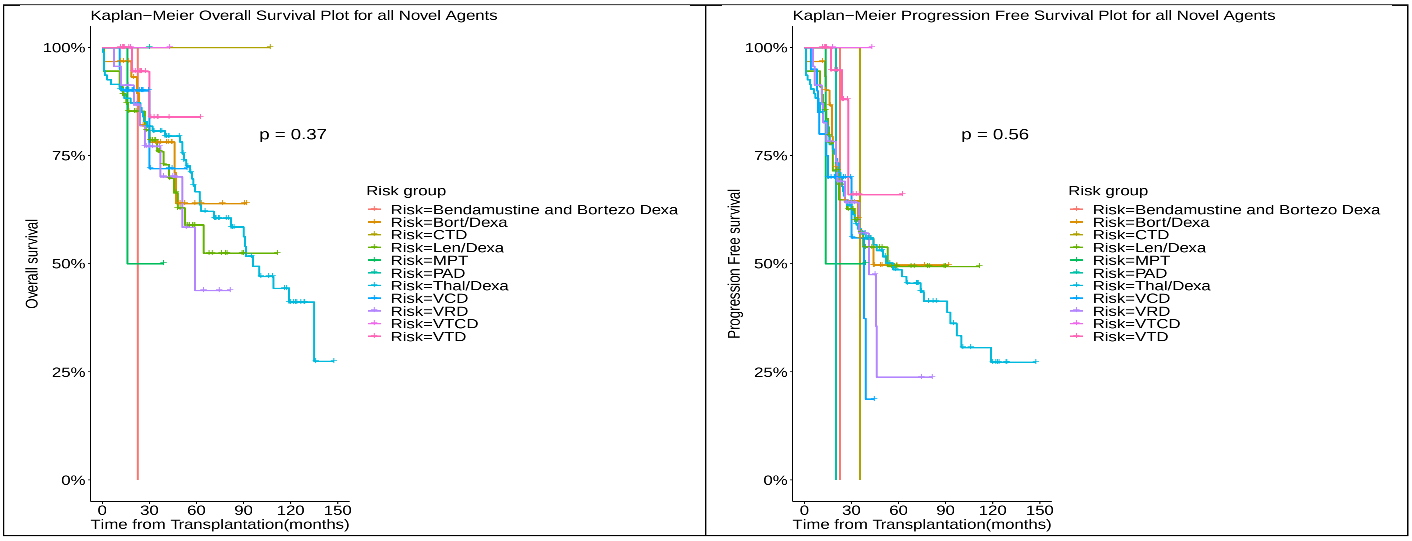

Figure S5: Comparing OS and PFS of various Novel agents

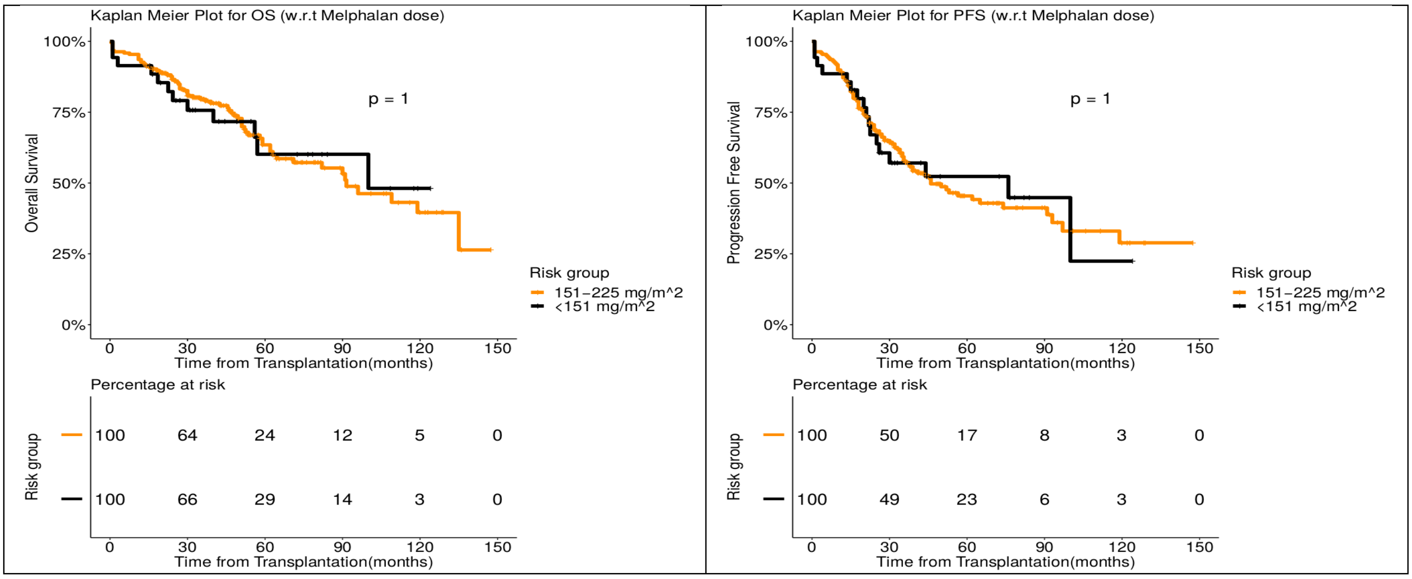

Figure S6: Comparing OS and PFS for different groups of Melphalan dosage shows that there is no significant difference between them. 218 patients received melphalan dosage $\geq 151mg/m^2$, and 35 patients received melphalan dosage < $151mg/m^2$.

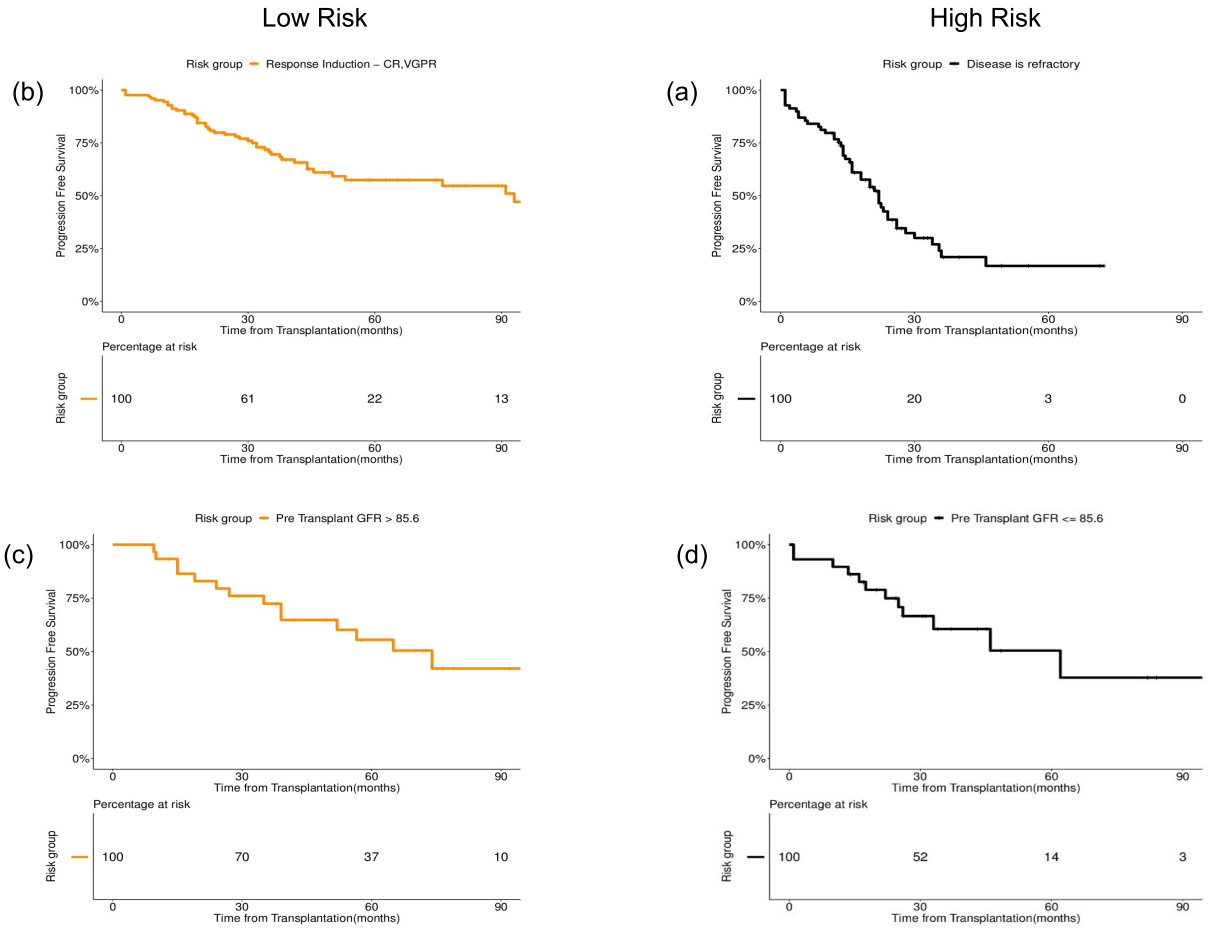

Figure S7: Progression Free Survival Curves for each node of FFT.
(a) If the relapse after remission status has 69 patients, 46 events, and a median survival time of 22 months.
(b) Complete Response (CR) and Very Good Partial Response (VGPR) to induction therapy has 125 patients, 47 events, and a median survival time of 93 months.
(c) Pre Transplant GFR > 85.6 has 30 patients, 15 events, and a median survival time of 74 months.
(d) Pre Transplant GFR <= 85.6 has 29 patients, 14 events, and a median survival time of 62 months.

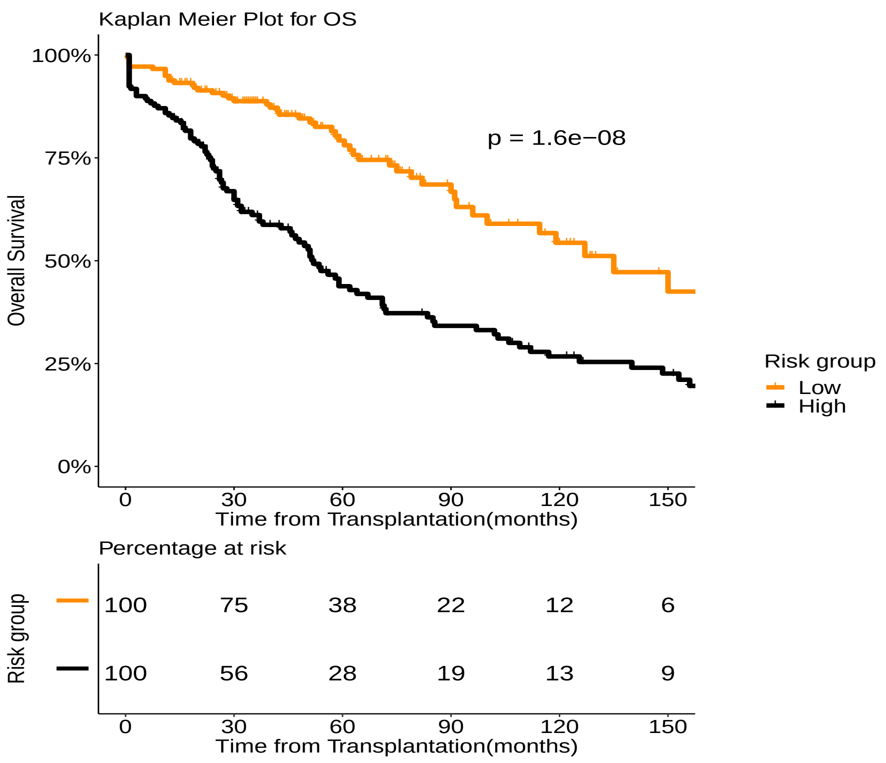

Figure S8: **Overall Survival (OS)** in all patients with multiple myeloma stratified by FFT rules. Median OS was more than 135 month (number of patients = 177, events = 49) for low risk group (orange colour), whereas it was 52 months (number of patients = 177, events = 106) for high risk group (black colour).

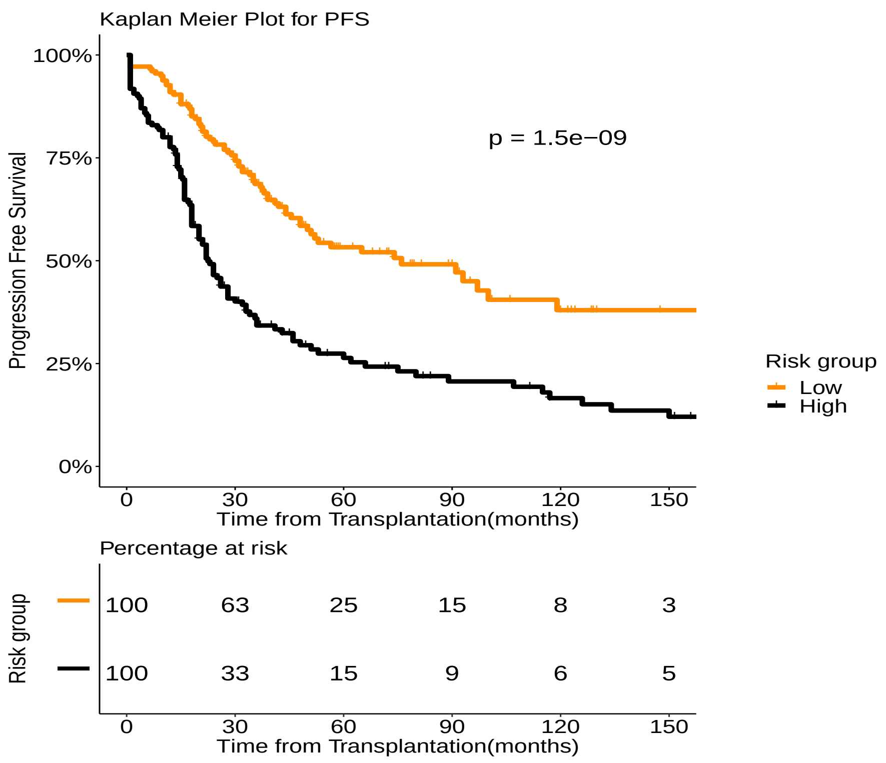

Figure S9: **Progression Free Survival (PFS)** in all patients with multiple myeloma stratified by FFT rules. Progression Free median OS is 76 month (number of patients = 177, events = 78) for low risk group (orange colour), whereas it is 22.5 months (number of patients = 170, events = 123) for high risk group (black colour).

REFERENCES

BladÉ, J., Samson, D., Reece, D., Apperley, J., BJÖrkstrand, B., Gahrton, G., et al. (1998). Criteria for evaluating disease response and progression in patients with multiple myeloma treated by high-dose therapy and haemopoietic stem cell transplantation. *British journal of haematology* 102, 1115–1123

Dimopoulos, M. A., Terpos, E., Chanan-Khan, A., Leung, N., Ludwig, H., Jagannath, S., et al. (2010). Renal impairment in patients with multiple myeloma: a consensus statement on behalf of the international myeloma working group. *Journal of Clinical Oncology* 28, 4976–4984

Durie, B. G., Harousseau, J., Miguel, J., Blade, J., Barlogie, B., Anderson, K., et al. (2006). International uniform response criteria for multiple myeloma. *Leukemia* 20, 1467

Durie, B. G. and Salmon, S. E. (1975). A clinical staging system for multiple myeloma correlation of measured myeloma cell mass with presenting clinical features, response to treatment, and survival. *Cancer* 36, 842–854

Greipp, P. R., Miguel, J. S., Durie, B. G., Crowley, J. J., Barlogie, B., Bladé, J., et al. (2005). International staging system for multiple myeloma. *Journal of clinical oncology* 23, 3412–3420

[Dataset] Podymow, T., Alam, A., Vasilevsky, M., Beauchemin, R., Shustik, C., and Sebag, M. (2010). A review of multiple myeloma patients on dialysis treated with high cutoff hemodialysis
